# Quantifying pesticide deposits and spray patterns at micro‐scales on apple (Malus domesticus) leaves with a view to arthropod exposure

**DOI:** 10.1002/ps.5136

**Published:** 2018-09-04

**Authors:** Joanna T Witton, Matthew D Pickering, Tania Alvarez, Melissa Reed, Gabriel Weyman, Mark E Hodson, Roman Ashauer

**Affiliations:** ^1^ Environment Department University of York York UK; ^2^ EcoRisk Solutions Ltd Norwich UK; ^3^ Chemicals Regulation Division, Health and Safety Executive York UK; ^4^ ADAMA Agricultural Solutions Ltd Thatcham UK

**Keywords:** fungicide, penconazole, orchard, residue analysis, spatial variation, water‐sensitive paper

## Abstract

**BACKGROUND:**

Pesticides used in commercial crop systems can adversely affect non‐target arthropod populations. The spatial distribution of pesticide residues is rarely studied at scales relevant to these populations. Here, we combine two methods for assessing pesticide spray deposits at spatial scales relevant to non‐target arthropods found in apple orchards. Pesticide residues were determined on individual apple leaves through conventional residue analysis; water‐sensitive paper was used to investigate spatial distributions in deposits at the micro‐scale. We also evaluated how accurately a digital image analysis program estimated pesticide residues.

**RESULTS:**

We found that mean pesticide spray coverage on water‐sensitive paper varied by up to 6.1% (95% CI 9.4%, 2.7%) within an apple orchard, and leaf residues varied by up to 0.95 (95% CI 0.54, 1.36) mg kg^−1^ within a tree. Leaf residues based on analytical chemistry were six times lower than pesticide deposition estimated through image analysis of water‐sensitive paper, although these correlated strongly. This correlation allowed estimation of actual residues by application of a correction factor.

**CONCLUSION:**

Our method demonstrates accurate estimation of pesticide deposits at the individual leaf scale through digital analysis of water‐sensitive paper and is a low‐cost, rapid alternative to conventional residue analysis techniques. © 2018 The Authors. Pest Management Science published by John Wiley & Sons Ltd on behalf of Society of Chemical Industry.

## 1 INTRODUCTION

Pesticides are a globally important tool in the control of pests and diseases in commercial crop systems;^1^, ^2^, ^3^ however, their use can adversely affect non‐target populations of arthropods, with both lethal and sublethal effects reported for a wide range of species.^4^, ^5^, ^6^, ^7^, ^8^ In recent years, the focus has turned more towards the sublethal effects on non‐target species;^9^, ^10^ this is partly due to the potentially prolonged exposure of both target and non‐target species to sublethal concentrations in real conditions.^11^

Sublethal effects such as reproduction changes are studied in non‐target arthropods as part of regulatory pesticide testing,^12^ and many studies have reported negative effects at recommended field rates.^4^, ^11^, ^13^ Regulatory studies typically apply pesticide products using total coverage of a test arena as they must consider the worst‐case exposure scenario. However, this causes a lack of realism in terms of the test exposure environment not matching the exposure realistically achieved within crop systems where exposure is patchy and varies spatially. Therefore, to bring realism to pesticide risk assessment, real exposure patterns must be considered at spatial scales relevant to the test species, either by modified toxicity and behaviour assays within laboratory settings or by computational modelling.

Various methods exist for assessing pesticide residue and exposure patterns, from the conventional method of taking samples from a field and extracting the residues in the laboratory,^14^ to the use of tracers such as fluorescent dyes 15 and artificial collectors such as water‐sensitive paper (WSP) 16 to investigate spray patterns such as deposition and coverage. Conventional residue testing, involving extraction, clean‐up and analysis steps, is usually conducted within the context of human exposure; for instance, to measure concentrations in food crops for dietary risk assessment.^14^, ^17^, ^18^ Some studies have also investigated residues on foliage,^19^, ^20^ a more relevant substrate in the context of non‐target arthropods (e.g. parasitoids and predators) in crop systems. These foliage methods typically work with large samples of several leaves with a mass of 10–20 g, but residues averaged across many leaves are not very relevant for predatory insects who are so small that heterogeneous exposure within one leaf is what matters. One study worked with individual leaves weighing ∼ 4.5 g,^21^ although apple leaves are typically smaller. A recently developed method is able to map pesticide residues on individual wheat leaves using MALDI‐MS,^22^ and this method shows promise but has limitations such as inaccuracies when working with dense spray coverage,^23^ access to such instruments and operational costs. Therefore, to investigate pesticide residues at spatial scales relevant to predatory arthropods, conventional residue testing methods need to be adapted to the scale of individual leaves.

One method applied widely to the study of pesticide spray patterns is the use of WSP.^24^, ^25^, ^26^ WSP is yellow card that is coated on one surface with bromophenol blue, which turns from yellow to blue on contact with water and retains this colour once dry, thus creating a stain.^27^ As such, these cards can be used to evaluate spray patterns as long as the pesticide spray mixture contains water.^28^ WSP has received a lot of attention from researchers who have developed methods for assessing spray quality manually using either a microscope, or by scanning and magnifying the image to evaluate cards by eye, although these methods are time‐consuming.^29^, ^30^, ^31^ More recent technology has allowed for the development of automated, computer‐based analysis of cards, with a number of programs available.^32^, ^33^, ^34^, ^35^ Studies comparing the efficacy of computer‐based programs against manual analysis of cards have found that a number of programs show strong correlations with manual analysis when comparing droplet diameters, volumes and counts computed from the same WSP cards.^36^ However, the authors found that there were differences of up to 10.4 times in droplet density values reported by different software packages, and thus suggest choosing one image analysis program and using it exclusively. Another comparison study found inconsistencies in relative droplet diameter values reported by three different programs, but also found that factors relating to 10th, 50th and 90th percentile diameters (*D*_V0.1_, *D*_V0.5_, *D*_V0.9_) were consistent.^37^

Although correlations between manual and digital assessment of WSPs were strong enough to prove that digital analysis successfully emulated manual analysis, this showed only that it was effective in assessing spatial patterns in spray such as droplet density and droplet size.^36^, ^37^ A further question is whether WSP can be used to determine pesticide residues. Through extraction and analysis of pesticide residues from WSP, a previous study demonstrated that droplet density and total mass of deltamethrin residues correlate well.^29^ One study suggested that pesticide deposition could be estimated through droplet analysis using a microscope, having studied the efficacy of the method with chemical tracers.^38^ In addition, liquid volumes derived through digital image analysis were consistent with microscopic droplet analysis.^30^

In summary, we know that WSP can be used to study spray distribution, and that digital image analysis of these paper samples can be quick, repeatable and consistent with manual analysis of samples.^34^, ^36^ We also know that pesticide residue analysis on an apple leaf substrate can be both accurate and precise.^19^, ^39^, ^40^ However, we do not know whether it is possible to accurately estimate pesticide residues using data derived from the analysis of pesticide‐exposed WSP, which would provide a low‐cost analysis method that allows for greater understanding of residues at micro‐spatial scales, such as within a single apple leaf.

In this study, we aimed to assess the accuracy of methods used to analyse spatially distributed pesticide residues. The first objective was to assess pesticide residues and spray patterns in an apple orchard at spatial scales relevant to orchard‐dwelling non‐target arthropods (e.g. leaf, tree, orchard scale). The second objective was to evaluate the potential for digital image analysis of WSP to be an effective alternative to conventional pesticide residue analysis for non‐target arthropod exposure estimation. To achieve this, patterns in data from both methods were compared.

## 2 MATERIALS AND METHODS

### 2.1 Orchard sampling

Field sampling was conducted in a commercial apple orchard in Cambridgeshire, UK in August 2015. The orchard had an area of 1.25 ha and contained 5‐year‐old dessert apple trees (*Malus domestica cv*. Braeburn) growing in rows 3.5 m apart and running from south‐west to north‐east in aspect. Trees were grown with a trellis support system with tree spacing within rows at 0.8 m. Trees were ∼ 3 m tall and in growth stage 8–9 according to the BBCH scale for pome fruit.^41^ Samples were collected following a routine spray application of the fungicide penconazole formulated as Topenco 100 EC (100 g L^−1^, Globachem NV, Sint‐Truiden, Belgium), an emulsifiable concentrate diluted in tap water for spray application at a rate of 450 mL diluted in 250 L water per hectare (final penconazole concentration = 0.18 g L^−1^). Penconazole has a photolytic degradation half‐life (λ) of 1.32–1.99 days; however, it is stable in darkness and hydrolytically stable at air temperatures of 50 °C for 7 days.^42^ Spray application was undertaken using a tower sprayer (Kirkland Tower Triprop Sprayer, Kirkland UK, Maidstone, UK), fitted with six each of Albuz ATR 80 yellow and green hollow cone nozzles (Solcera, Moissy Cramayel, France) working at a spray pressure of 6 bar. Crop spraying commenced at 9.40 am in overcast conditions at an air temperature of 19.8 °C with wind speeds of 0.54 m s^−1^ (60 s average) and 1.97 m s^−1^ (60 s maximum). Samples were collected once pesticide residues were dry, after ∼ 1 h.

**Figure 1:**
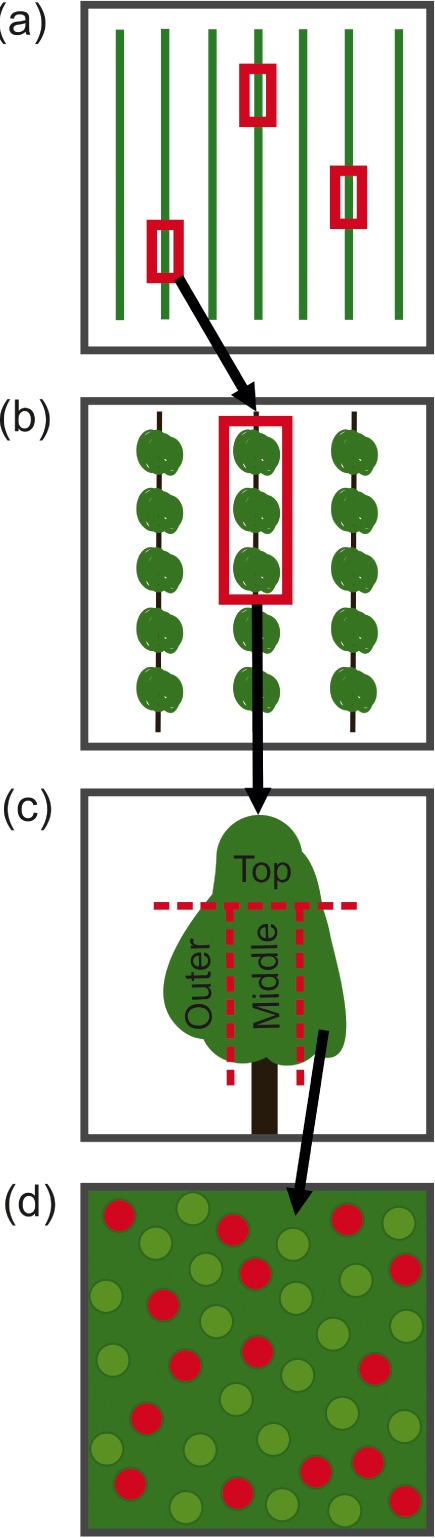
Schematic of the nested orchard sampling design showing the four spatial scales used for apple leaf residue and water‐sensitive paper coverage analysis. Each spatial scale is represented: (a) patch locations, (b) one patch comprising three trees, (c) one tree comprising three zones, and (d) several samples within one zone.

An experimental design with nested spatial scales was established for the orchard sampling, similar to one outlined previously.^43^ Three rows within the orchard were selected, with one patch (A, B and C) per row. Each patch contained three consecutive trees and was located away from row ends to ensure pesticide spray was representative. Each tree was then split into three zones: top, middle and outer, with the top portion starting ∼ 2 m above ground (Fig. 1).

### 2.2 Residue sample collection

Prior to the scheduled pesticide application, 12 WSP cards measuring 26 × 76 mm (Syngenta, Basel, Switzerland) were placed in the middle and outer zones in each tree across the three patches; these were attached to the upper leaf surface using a small bulldog clip at the stem to minimize the impact of the additional weight. We were unable to access the top tree zone for placement of WSP cards due to health and safety constraints. After allowing 60 min for the pesticide spray to dry, the WSP cards were collected and stored at ambient air temperature in sealed plastic bags within an opaque box until analysis.

Five apple leaves were collected from trees prior to pesticide application to act as blank samples for residue analysis. Following pesticide application and a drying period of ∼ 60 min, 45 apple leaves were sampled from each tree in patch A using telescopic secateurs, with 15 leaves from each of the three tree zones in each tree. Leaf samples were stored individually in centrifuge tubes in the dark at 10 °C in the field before being transferred after 24 h to a − 20 °C freezer until analysis.

### 2.3 Leaf sample extraction and clean‐up

Our method was adapted from one for extracting pesticide residues from bulk samples of leafy vegetables (10 g),^44^ to the extraction of individual leaves by adjusting extractant volume to be of a similar ratio to the original method. The sampled leaves had a mean mass of 0.57 (95% CI 0.53, 0.62) g. Leaf samples were removed from the freezer and allowed to defrost at ambient temperature. Weight and upper leaf area were determined for each leaf before they were extracted, with leaf area determined by scanning and image analysis in ImageJ (v. 1.38x, NIH, Bethesda, MD, USA). Each leaf was cut into smaller pieces and returned to the centrifuge tube for extraction with 10 mL acetonitrile. Samples were homogenized in an ultrasonic bath for 10 min. An 8.5‐mL aliquot of the extraction solution was then transferred to a glass test tube and concentrated to 1–2 mL under a gentle stream of nitrogen gas using a heated sample concentrator (Techne Dri‐Block DB‐3; 40 °C, N_2_ flow rate of 8 L min^−1^).

For sample clean‐up, a solid‐phase extraction (SPE) cartridge (Supelclean ENVI‐Carb‐II/PSA 500 mg/500 mg, 6 mL size) was conditioned with 3 mL acetonitrile/toluene (3:1 v/v). The acetonitrile sample extract was then loaded onto the cartridge and the retained pesticide was eluted slowly with 3 mL acetonitrile/toluene (3:1 v/v). The final eluate was then evaporated to dryness and reconstituted in 1 mL acetonitrile. The sample was mixed using a vortex mixer (25 000 rpm, 5 s) before transfer to an amber autosampler vial for analysis via gas chromatography–mass spectrometry (GC–MS).

To compare leaf residue data from analytical chemistry with residues estimated from image analysis, penconazole residue data were converted so that residues were based on leaf upper surface area (Eqn (1)). It was not within the scope of this study to determine what proportion of pesticide spray lands on the upper and lower leaf surfaces, therefore the leaf residue values assume all residue was present on the upper leaf surface. Previous studies have investigated differences in upper and lower leaf surface residues.^39^

(1) $$R_{\text{area}}=\frac{R_{\text{leaf}}}{A}$$

*R*_area_ denotes residue based on leaf surface area in µg cm^−2^, *R*_leaf_ denotes residue detected on a leaf sample in µg, and *A* denotes leaf area in cm^2^.

### 2.4 Gas chromatography–mass spectrometry

Penconazole residues were determined using a Clarus 680/600C GC–MS (PerkinElmer, UK) fitted with an Elite‐5MS fused silica capillary column (L 30 m × 0.25 mm i.d. × 0.25 µm film thickness; PerkinElmer). Samples were prepared in acetonitrile for analysis and 1 µL injected via a split–splitless injection port operated in splitless mode (splitless time 1 min; injector temperature 250 °C). The oven was programmed from an initial temperature of 50 °C (hold 1 min) to 300 °C at a rate of 20 °C min^−1^ where it was held for 3 min. Helium (99.999% purity) was used as the carrier gas at a flow rate of 1 mL min^−1^. The MS was operated in electron ionization (EI) mode with an ionization energy of 70 eV, source temperature of 180 °C and inlet line temperature of 240 °C. Data were acquired in selected ion monitoring (SIM) mode at *m*/*z* 159 and 248 (dwell time 100 ms), used for quantification of penconazole.^44^ The solvent delay was set to 4 min and the total run time was 16.5 min. Penconazole eluted with a retention time of 11 min. Instrument control, data acquisition and processing was by Turbomass software v5.4.1617 (PerkinElmer).

### 2.5 Analytical method development

To determine the accuracy of the GC–MS method, apple leaves washed in deionized water were spiked with known quantities of penconazole analytical standard (Sigma Aldrich, Gillingham, UK) in acetonitrile at five concentrations ranging from 0.1 to 4 µg per leaf, a range that covered all field residues determined in this study. These leaves were subject to extraction and SPE using the method outlined previously. Penconazole recovery was calculated along with the coefficient of variation (CV). Method precision was determined by extracting five apple leaves spiked with 2 µg penconazole, and was expressed as CV. In a further step to validate the experimental method, a storage stability study was conducted in which 10 washed apple leaves were each spiked with 2 µg penconazole. One set of five leaves was extracted and analysed once residues were dry, and one set of five leaves was stored at −20 °C after drying for a total of 37 weeks before being defrosted, extracted and analysed. The penconazole recovery rate was determined along with CV.

Recoveries were in the range 74–119% (mean 90%; *n* = 21), with CV of 5.9–24.9% (mean 11.9%) for all standard concentrations. Precision, calculated from five 2 µg mL^−1^ samples, was 7.9% and within the acceptable CV limit of 20%.^45^ Our storage stability study showed that recovery was in the range 83–110% (mean 99%) after 37 weeks.

Analytical limit of detection (LOD) and limit of quantitation (LOQ) were calculated using signal‐to‐noise ratios of 3:1 and 10:1 respectively,^46^ determined for spiked apple leaves. LOD and LOQ for penconazole were 0.08 and 0.26 mg kg^−1^ respectively. No field samples had detected residues that were below the LOQ, including the controls collected before the spray event.

### 2.6 Spray pattern analysis

Following a review of the various image analysis packages available, DepositScan, a freely available droplet analysis program was selected for use in this study.^34^ Individual WSP cards were scanned on a flatbed scanner (Canon CanoScan 9000F) at a resolution of 600 dpi as greyscale bitmap images and cropped and converted to GIF file type using Irfanview v. 4.4. Several factors were determined by DepositScan including spray coverage (percentage of target covered), spray density (droplets cm^−2^) and liquid deposition (µL cm^−2^). The smallest droplet diameter that can be detected by DepositScan is 17 µm;^34^ however, in this study the smallest droplet diameter was 52.8 µm. These were analysed at the various spatial scales sampled (within tree, between tree, within orchard). Volume median diameter (VMD) is a common measure when describing droplet sizes; however, this is easily skewed by factors such as a few large droplets amongst a mostly small droplet pattern;^28^ thus this metric was omitted.

It is possible to calculate estimated pesticide deposits on apple leaves using data from DepositScan, if the concentration of pesticide in the spray tank mixture is known (Eqn (2)).

(2) R=C×D

*R* denotes active ingredient residue in µg cm^−2^; *C* denotes concentration of active ingredient in spray tank mixture in µg µL^−1^; *D* denotes the liquid deposition value calculated by DepositScan in µL cm^−2^.

### 2.7 Testing DepositScan

To test the way in which DepositScan calculates factors such as deposition, a number of tests were designed involving computer‐generated images of ‘stains’, with all images generated using Microsoft Paint. Stains on WSP are larger than the area covered by the initial droplet due to the solution spreading.^28^ As WSP absorbs and expands the aqueous portion of a pesticide spray, it has been suggested that the measurement of WSP stains can overestimate the liquid deposition,^47^ as the stains created appear larger than the initial droplet. However, through using a spread factor, this error can be accounted for.

To calculate the initial droplet diameter from which droplet volume can be derived, a spread factor was applied – DepositScan uses a formula where a single factor is applied to the stain area (Eqn (3)).^34^ An alternative spread factor calculation exists based upon stain diameter, with the spread factor varying based upon stain diameter. Documentation providing further information about WSP reports a range of spread factors, with spread factor values increasing as stain size increases (Table 1). The factors were determined using water sprayed at 20 °C and 40% relative humidity, though the authors state that pH and relative humidity have no effect.^48^

(3) $$d=\mathrm{SF}\times A^{0.455}$$

**Table 1:** Variable spread factor values determined based on water and used as an alternative to DepositScan's built‐in spread factor calculation 28, 48

Stain diameter of droplet (µm)	Spread factor	Droplet diameter (µm)
100	1.7	58.8
200	1.8	111.1
300	1.9	157.9
400	2.0	200.0
500	2.1	238.1
600	2.1	285.7

*d* denotes droplet diameter in µm; *SF* denotes the spread factor (the DepositScan default used here is 1.06); *A* denotes the spot area calculated by DepositScan in µm^2^.

To apply this variable spread factor, the stain diameter was calculated from the stain area derived from DepositScan (Eqn (4)), and from this the droplet diameter was calculated using Eqn (5)) and applying the relevant spread factor from Table 1; for example, the spread factor for a droplet with a stain diameter in the range of 301–400 µm would be 2.0.^48^

(4) ds=Aπ×2

*d*_s_ denotes stain diameter in µm and *A* denotes spot area calculated by DepositScan in µm^2^.

(5) $$d=\frac{d_{s}}{\mathrm{SF}}$$

*d* denotes droplet diameter in µm; *d*_s_ denotes stain diameter in µm and *SF* denotes the spread factor value from Table 1 that applies to *d*_s_.

To assess whether calculated deposition changed when different spread factors were applied to stain size data, we generated 33 artificial circular ‘stains’ with diameters in the range 95.5–1438 µm. These were analysed using DepositScan, and calculated deposition values derived from DepositScan's own calculation and from the application of the varied spread factor were compared. To determine deposited volume, each droplet diameter was converted to volume.

We also quantified the effect of droplets of equal size touching each other. The software creators state that DepositScan cannot differentiate between droplets that overlap, and so the software makes the assumption that two touching droplet stains are one single deposit.^34^ To assess whether this affected the estimation of deposition, the same artificial stains from the spread factor tests were used, with two images at each stain diameter analysed: one containing two stains of equal size that did not touch, and one containing two stains that touched at one side, but did not overlap. This meant that an image with two droplets each of 96 µm diameter had a horizontal diameter of 192 µm, but still measured 96 µm at the longest vertical point, or vice versa.

To calculate the droplet volume for each droplet, DepositScan uses Eqn (5)), and the sum of each volume is reported as deposition expressed as µL cm^−2^.^34^ In this test, we compared the deposition value in µL per image for each image pair.

### 2.8 Statistical analysis

Analyses were undertaken using GraphPad Prism (v. 7.01, GraphPad Software Inc., La Jolla, CA, USA). All residue and WSP data were initially tested for normality using the D'Agostino–Pearson normality test;^49^ the Brown–Forsythe test for equality of variances was also used to determine the best test for data sets.^50^ Data were analysed using one‐way analysis of variance (ANOVA) with Tukey's HSD test for multiple comparisons. When comparing exposure between trees, there was no grouping so all nine trees were compared with each other, with trees 1–3 from patch A, trees 4–6 from patch B and trees 7–9 from patch C. Data sets comparing exposure between two tree zones were tested for normality using D'Agostino–Pearson, and then analysed using the unequal variances *t*‐test (also known as Welch's *t*‐test), chosen for its ability to handle unequal population variances.^51^

Data relating to testing DepositScan were tested for normality as above, but because of non‐normal distributions in both tests, data were analysed using the Wilcoxon matched‐pairs ranked test. Regression analysis was performed to investigate whether leaf residues based on leaf area were comparable to residues based on leaf mass.

## 3 RESULTS

### 3.1 Spray pattern analysis

Mean pesticide coverage of the WSP surface was 16.3% (95% CI 15.1, 17.5) across all samples (Table 2; *n* = 215). Mean surface coverage in patch A was 20.1%, 14.9% in patch B (mean difference A vs B 5.2%; 95% CI 8.5, 1.8; *P* = 0.0009), and 14% in patch C (mean difference A vs C 6.1%; 95% CI 9.4, 2.7; *P* < 0.0001).

**Table 2:** Pesticide spray coverage, expressed as proportion of target covered by spray, and spray density, expressed as the number of droplets in an area , determined from water‐sensitive paper cards set within apple trees

	Target covered (%)						Spray density (droplets cm^−2^)				
			95% CI					95% CI			
	*n*	Mean	Lower	Upper	CV (%)	*P*‐value	Mean	Lower	Upper	CV (%)	*P*‐value
Within orchard											
Patch											
A	70	20.1	18	22.1	43.1	<0.0001	132.8	124.6	141	25.9	<0.0001
B	73	14.9	12.8	17	60.6		101.4	91.8	111	40.5	
C	72	14	12.2	15.8	53.7		126.1	115.3	136.9	36.3	
Within patch											
Tree											
1	24	22.8	18.6	27	43.9	0.13	133.2	120.2	146.2	23.1	0.95
2	23	19.4	15.6	23.3	46.2		130.9	113	148.8	31.6	
3	23	17.8	15.2	20.4	33.7		134.2	120.5	147.9	23.6	
4	24	15.1	10.9	19.3	65.9	0.85	101	85.34	116.6	36.7	0.88
5	24	15.6	12.2	18.9	50.4		104.5	88.7	120.4	36.7	
6	25	14.1	10.2	18	67.2		98.6	78.1	119.1	49.2	
7	24	14.6	11.9	17.3	43.3	0.87	121.9	105.5	138.3	31.8	0.86
8	24	13.5	10	16.9	60.4		128.7	106.5	150.9	40.1	
9	24	13.9	10.4	17.4	59.2		127.8	108	147.5	36.6	
Within tree											
Zone											
Middle	106	15.3	13.7	17	55.8	0.12	115.3	106.8	123.8	38.2	0.12
Outer	109	17.2	15.5	18.9	52.2		124.4	116.6	132.2	33.1	

Data are expressed as mean coverage with 95% confidence intervals (CI), showing differences within the sampled orchard, within patches and within trees. Coefficient of variance (CV) is also presented. *P*‐values for within‐patch variance are for each group of three trees that made up each patch, e.g. patch A is comprised of trees 1–3.

When analysing variance between all nine trees, the mean difference in pesticide coverage was 3.4% (*P* = 0.002). Tree 1 received the greatest pesticide coverage (Table 2) and significantly greater coverage than tree 4 (mean difference 7.7%; 95% CI 15.3, 0.03; *P* = 0.048), tree 6 (mean difference 8.7%; 95% CI 1.1, 16.3; *P* = 0.012), tree 7 (mean difference 8.2%; 95% CI 0.53, 15.8; *P* = 0.026), tree 8 (mean difference 9.3%; 95% CI 1.66, 16.95; *P* = 0.026) and tree 9 (mean difference 8.9%; 95% CI 1.23, 16.52; *P* = 0.026). Mean coverage in the middle zone was 15.3% and 17.2% in the outer zone (*P* = 0.12).

Spray density averaged 120 (95% CI 114, 126) droplets cm^−2^ across all samples (Table 2; *n* = 215). On average, density varied significantly by 4.45 droplets cm^−2^ between patches (*P* < 0.0001), although in contrast to the trend shown in coverage, spray density was 31.4 (95% CI 47.5, 15.3) droplets cm^−2^ higher in patch A than patch B (*P* < 0.0001), and 24.7 (95% CI 8.7, 40.7) droplets cm^−2^ higher in patch C than patch B (*P* = 0.001). Spray density did not significantly vary within patches (Table 2). There was also no significant difference in spray density between tree zones, which is consistent with coverage trends.

### 3.2 Leaf residues

Penconazole residues found on apple leaves in patch A were between 0.35 and 6.56 mg kg^−1^ (Table 3). The mean difference in residues between trees was 0.22 mg kg^−1^ (*P* = 0.18). By contrast, residues varied within trees by 0.63 mg kg^−1^ on average (*P* < 0.0001), with residues in the top tree zone 0.95 (95% CI 0.54, 1.36) mg kg^−1^ higher than in the middle zone (*P* < 0.0001) and 0.63 (95% CI 0.22, 1.04 mg kg^−1^) mg kg^−1^ higher in the outer zone than in the middle zone (P = 0.0005). The mean difference between top and outer zones of the trees was 0.32 (95% CI −0.08, 0.74) mg kg^−1^ (*P* = 0.657).

**Table 3:** Penconazole residues in apple leaves from patch A, expressed as mean residue with 95% confidence intervals (CI), split by tree and tree zone

		Penconazole residue in apple leaf (mg kg^−1^)				
			95% CI			
	*n*	Mean	Lower	Upper	CV (%)	*P*‐value
Patch						
A	135	2.28	2.09	2.47	48.31	—
Tree						
1	45	2.03	1.7	2.36	63.4	0.18
2	45	2.44	2.08	2.79	40.8	
3	45	2.37	2.08	2.67	47.5	
Zone						
Top	45	2.72	2.42	3.01	43.2	<0.0001
Middle	45	1.76	1.55	1.98	65.1	
Outer	45	2.39	2.17	2.61	41.8	

Coefficient of variance (CV) is also reported.

### 3.3 Comparing residue analysis methods

**Figure 2:**
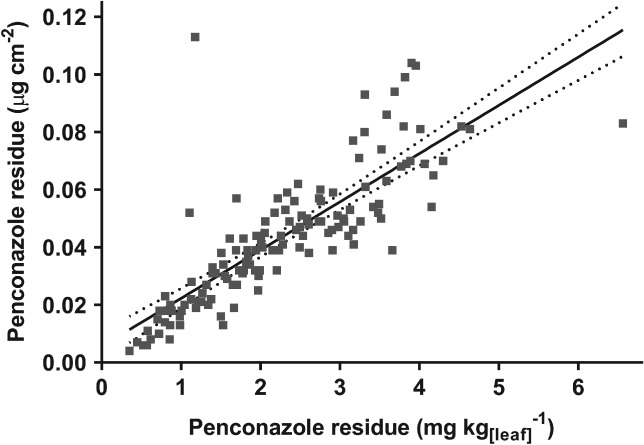
Penconazole residues derived from GC‐MS based on leaf mass (horizontal axis) and whole‐leaf upper surface area (vertical axis). Solid trend line shows regression with 95% confidence bands (dotted lines). Regression: *Y* = 0.01675 × *X* + 0.005528. *R* ^2^ = 0.65; *P* < 0.0001; *n* = 135.

To compare leaf‐derived penconazole residue data with penconazole deposits estimated from WSPs by DepositScan, residue data had to be converted to a comparable unit, so the following data are expressed as µg cm^−2^. Because leaf residue measurements were undertaken only on leaves from patch A, WSP data from patches B and C were omitted to ensure data were comparable. Similarly, leaf residue data from the top tree zone were also omitted. Any residue estimates derived from WSP samples with coverage > 30% were also omitted because volume estimates become inaccurate above this point.^34^ Regression analysis suggests that area‐based leaf residue values correlate well with the mass‐based leaf residue values (*R* ^2^ = 0.65; *P* < 0.0001; Fig. 2). Two main outliers that deviate far from the 95% CI boundary represent samples with lower leaf mass, or smaller leaf areas than average.

**Figure 3:**
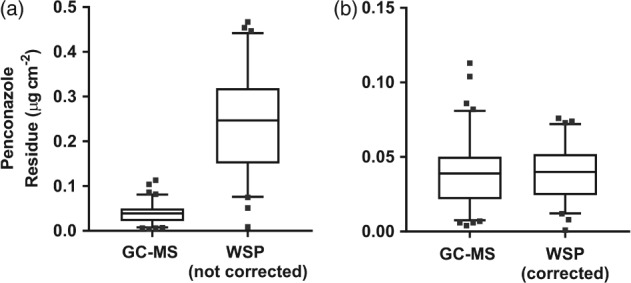
Comparison of penconazole residue values based on surface area derived from leaf residue samples analysed via GC‐MS and water‐sensitive paper samples analysed via DepositScan image analysis with no correction factor (a) and with a correction factor applied to individual data points (b). The horizontal middle, lower and upper lines within each box indicate mean, 25th and 75th percentiles; caps at the top of the vertical lines indicate the 5th and 95th percentiles; dots depict extreme data points (i.e. values less than 25th percentile − 1.5 × interquartile distance, or greater than 75th percentile + 1.5 × interquartile distance). *n* = 90; 61.

Penconazole residues based on leaf surface area averaged 0.039 (95% CI 0.035, 0.044) µg cm^−2^; the WSP‐derived values averaged 0.24 (95% CI 0.21, 0.27) µg cm^−2^ (*P* < 0.001; Fig. 3a). The mean difference between the leaf residue and WSP estimate was 0.198 (95% CI 0.17, 0.23) µg cm^−2^ (*P* < 0.001). To adjust the WSP mean penconazole deposit to that of the apple leaf residues, an empirical correction factor of 0.1625 was applied to the WSP data (Eqn (6)), and this successfully adjusted the mean penconazole deposit to 0.039 (95% CI 0.035, 0.044) µg cm^−2^ (Fig. 3b). The 0.00013 (95% CI −0.006, 0.006) µg cm^−2^ mean difference between mean corrected WSP residue values and leaf residue values was not significant (*P* = 0.97).

(6) $$R_{\text{corrected}}=R\times \mathrm{CF}$$

*R*_corrected_ denotes corrected penconazole residue in µL cm^−2^; *R* denotes penconazole residue on WSP in µL cm^−2^; *CF* denotes the correction factor of 0.1625.

### 3.4 Testing DepositScan

**Figure 4:**
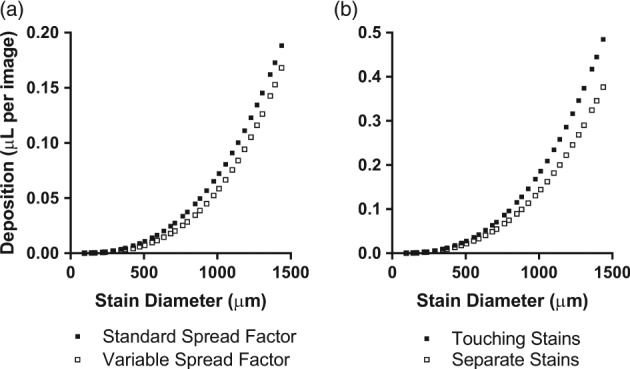
(a) Deposition values calculated from the same artificial stain using the default DepositScan spread factor and an alternative spread factor that varies based on stain diameter (*n* = 33). (b) Deposition values calculated by DepositScan based on whether two artificial circular stains of the same size touch (*n* = 33).

When comparing different spread factor calculations, mean droplet volume for the DepositScan spread factor was 0.055 (95% CI 0.035, 0.075) µL per image, 19.6% higher than the average droplet volume from the varied spread factor of 0.046 (95% CI 0.028, 0.064) µL (*P* < 0.0001; ratio‐paired *t*‐test). Deposition ranged from 0.00009 to 0.1882 µL when calculated using the DepositScan spread factor, and from 0.00009 to 0.1681 µL when calculated with the varied spread factor (Fig. 4a). When assessing whether deposition is overestimated when two droplets touch compared with when there is no contact, the respective mean deposition volumes were 0.14 (95% CI 0.09, 0.19) µL and 0.11 (95% CI 0.07, 0.15) µL, thereby showing deposition was overestimated on average by 22.3% when the droplets were touching (*P* < 0.0001; Fig. 4b).

## 4 DISCUSSION

### 4.1 Comparison of trends in leaf residue and water‐sensitive paper data

Measurements derived from WSP analysis indicated there was a significant average difference of 28% in spray coverage and 20% difference in spray density between patches within the same orchard (Table 2). This is in contrast to findings from a study in which a zinc tracer was sprayed in an apple orchard where no significant differences between plots within the same orchard were observed.^43^ These contrasting findings could be due to differences in weather conditions during the study, spray systems or settings, the methodology to identify spray coverage and density, sample numbers, tree type, tree spacing, etc. and highlight the problems with interstudy comparisons. However, the nested sampling design used in our study enables detailed analysis of sources of variation within a single orchard that can be compared with findings in other studies. When studying trends within orchard patches, we found no significant differences in spray coverage or density; this was also the case within patch A for apple leaf residues. When focusing on patch A, tree 1 displayed the highest spray coverage, the second highest spray density and the lowest leaf residue. This suggests that trends differ depending on the measurement analysed. Although the trees chosen for this study were uniform in height, growth habit and management, such differences in spray patterns between trees have previously been justified by tree architecture, in particular variability in leaf position.^43^ This seems to be consistent with our findings.

In considering variance within trees, spray cover, density and leaf residues were all higher in the outer zone than in the middle zone; leaf residues were higher in the top tree zone than in the middle (Table 3). The trend of outer tree sections receiving more residue than the centre is consistent with trends previously reported in a similarly designed apple orchard study using EDTA chelates of various metals as tracers 52 and also in the zinc EDTA chelate tracer study,^43^ suggesting that our study method was a successful model of real pesticide exposure when compared with other studies. Between‐tree variation for measures based on WSP such as spray coverage and spray density showed consistent but different trends to the actual residue analysis, and all were not significant, suggesting natural variation was responsible for observed differences. However, within a single patch of trees, spray pattern measurements showed no significant trends between trees. With observed trends differing at different spatial scales, we suggest an unmeasured factor may have an impact, such as variable proximity of the crop sprayer to trees.

When considering within‐tree differences, leaf residue trends were consistent with spray coverage and density trends, with values in the outer zone higher than in the middle zone, although effect size varied: leaf residues were 36% higher in the outer zone, a significant difference, whereas spray coverage and density showed non‐significant differences where coverage was 12% higher and density was 8% higher in the outer zone. This suggests that, although WSP analysis accurately estimates overall trends in within tree differences, effect size would be underestimated.

### 4.2 Comparing residue analysis methods

Overall, penconazole deposits estimated through image analysis of WSP were more than five times higher than residues determined through GC–MS analysis of exposed apple leaves (Fig. 3b). However, there was also a significant difference in variances, with overall CV higher for the leaf residues measured with GC–MS (52%) than it was for the WSP deposits (44%; *F*‐test; *P* < 0.001). This suggests that trends derived from WSP analysis are less affected by random variation. Our analytical method validation demonstrated that the penconazole extraction method was within validation guidelines;^45^ additionally, the storage stability study demonstrated minimal penconazole losses of 1% on average over time. Therefore, our findings suggest that DepositScan consistently overestimated penconazole residues when compared to the leaf residues.

The source of overestimation could come from the farmer's dilution of pesticide product when preparing spray tank mixtures, as varying precision in the preparation could provide a source of error. The overestimation could also come from the spray tank mixture behaving differently on the WSP surface in comparison to water, which is used by the paper manufacturer in the preparation of varied spread factors.^48^ Additionally, many factors relating to DepositScan's estimation of residues. First, the software assumes droplet stains are circular, and is not capable of identifying droplets that are overlapping,^34^ and this can cause an overestimation of spray deposition. Second, when coverage is > 30%, spray density and deposition are potentially inaccurate,^34^ as droplets are more likely to be touching when there is a high coverage value. In this study, 12% of all WSP cards displayed coverage > 30%.

**Figure 5:**
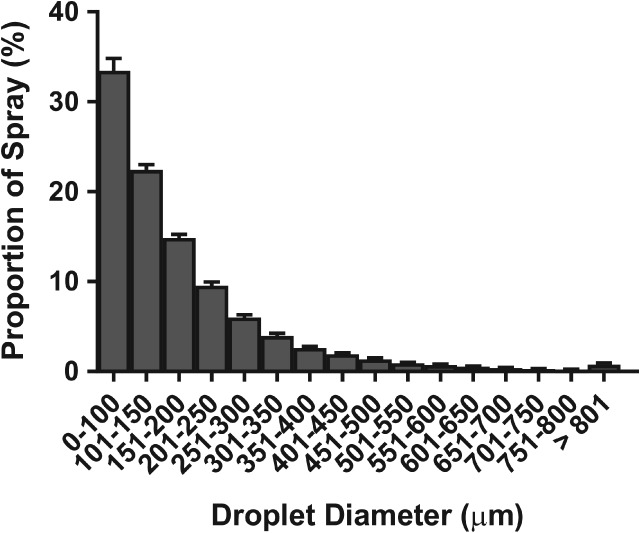
Distribution of droplet diameters as a function of spray proportion on water sensitive papers from patch A. Bars show 95% confidence intervals (*n* = 70).

A comparison of spread factors found that the factor used by DepositScan produced deposition values that were on average 11.5% higher than the deposition calculated from the varied spread factor. Table 4 shows how the two different spread factors would calculate droplet diameter from six hypothetical stain sizes, and displays a range of differences, from 5.8% at 200 µm diameter, to 12.3% at 500 µm diameter. Although no studies have investigated whether image analysis programs overestimate deposition, one study showed that, when compared with manual analysis of WSP, DepositScan overestimated droplet density by 89% when dealing with fine droplets.^36^ From further analysis of WSP samples from patch A, on average 33% of all droplets were determined to be ≤ 100 µm in diameter, with > 70% of all droplets measuring < 200 µm in diameter (Fig. 5). This strong skew suggests that DepositScan's overestimation of droplet density associated with fine droplets is a contributing factor in this study, and could also affect other calculated factors.

**Table 4:** The effect of two different spread factors on six stain diameter sizes. The spread factors were determined on water droplets

	Droplet diameter (µm)		
Stain diameter (µm)	DepositScan spread factor	Varied spread factor	Difference (%)
100	62.74	58.82	6.25
200	117.90	111.11	5.76
300	170.51	157.89	7.40
400	221.54	200.00	9.72
500	271.42	238.10	12.28
600	320.40	285.71	10.83

One other reason for the overestimation could be that wetting agents within the pesticide formulation could cause the droplet to spread further on impact with a surface due to an altered surface tension, therefore creating a larger stain than that created by water alone. The aforementioned variable spread factors were developed based on droplets of water, as opposed to pesticide mixtures,^48^ which in theory would produce smaller stains for a droplet of the same volume. Finally, when a pesticide is sprayed onto a plant some of the active ingredient might translocate into the plant, thus leading to lower residue values on the plant surface. This may be another source of the overestimation seen when analysing WSP.

Despite the overestimation of residue from WSP, it is possible to use WSP and DepositScan to estimate pesticide residues with a correction factor. However, this correction factor might only apply to the penconazole formulation and application regime in this study, and more work is necessary to determine whether a single correction factor would work for all spray situations and pesticide formulations, or whether correction factors would be specific to formulations (e.g. due to different wetting agents, application concentrations).

Digital image analysis of WSP to estimate pesticide deposits provides a time‐ and cost‐effective high‐throughput method of studying pesticide residues in agricultural systems. At the time of writing, WSP cost under £40 for 50 pieces, and digital analysis of each sample can be completed in a matter of minutes. By comparison, conventional residue analysis methods involve pesticide extractions that can take over a day to complete, with chromatographic analysis time requirements varying based on method and retention times for the chosen study compounds. The analysis cost can be prohibitive due to the use of SPE techniques and the operation of analytical equipment. Therefore, the time and cost savings of using WSP are attractive as they allow for large sample numbers in study designs; WSP also allows for examination of pesticide spray patterns at a fine spatial scale, something that is not currently widely undertaken by conventional residue analysis.

## 5 CONCLUSIONS

Pesticide spray patterns can differ within an orchard, between and within trees during a single pesticide spray event. Trends showing differences in coverage, spray density and residue within trees are important for understanding the exposure patterns that tree‐dwelling arthropods are subjected to in commercial apple orchards. Together with the micro‐scale exposure patterns derived from the WSPs, these data will inform future experiments looking at effects of pesticide exposure on their behaviour.

Through the study of pesticide spray patterns, we have been able to demonstrate that WSP could act as a replacement for conventional residue testing by offering a fast method of estimation, but the sources of uncertainty need to be fully understood. Water‐sensitive paper is a highly effective tool for analysing differences in spray patterns and allows for large sample numbers and rapid estimation of pesticide deposits on a surface. It also provides data on spray density and coverage that residue analysis cannot; however, if residue values are an important factor in the study of an untested compound then conventional residue analysis techniques should still be considered. Once the correlation between WSP data and conventional residue analysis is established for a pesticide product, with an adjustment factor if necessary, large numbers of WSP samples can be deployed for high throughput exposure analysis. Further work should explore whether pesticide residues derived from WSP correlate well with other pesticide formulations, and whether different correction factors are necessary for different pesticide formulations or situations.
